# Caught between financial and psychological distress: a call for integrated financial readiness and community-based mental health interventions in the U.S.

**DOI:** 10.3389/fpubh.2026.1931063

**Published:** 2026-10-26

**Authors:** Sophia N. Ryder, Rachel Kovacs, Xu Zhang, Traci Marciniak, Adam D. Brown

**Affiliations:** 1 The New School, New York, NY, United States; 2 Department of Economics, Farmingdale State College, Farmingdale, NY, United States; 3 Arcamind Global Institute for Mental Health Solutions, Duluth, GA, United States; 4 Department of Psychiatry, New York University, New York, NY, United States

**Keywords:** adaptation, community-based, financial wellbeing, global mental health, task-sharing

## Abstract

Financial stress and mental health disorders impact much of the general population globally. In addition, studies show that mental health concerns and financial stress are highly interconnected. On the one hand, mental health concerns and substance use disorders have been linked to factors associated with financial hardship, such as debt and insecurity. On the other, financial stress has been linked to the onset and maintenance of mental health disorders. Given the gaps in access to services that address mental health disorders and financial hardship, community-based models have been developed to provide relevant services. To date, however, community-based models supporting mental health and financial stress address these issues separately, with few strategies taking an integrative approach. The adaptation of community-based models for mental health and financial readiness represents an important strategy for developing new interventions to understand the dynamics between mental health concerns and financial stress. We propose the need for research and implementation efforts directed at integrating community-based financial and mental health interventions.

## 1 Introduction

It is well documented that there are strong relationships between financial stress, the physical and mental health symptoms caused by difficulties managing finances (1) and mental health (2, 3). Financial hardship contributes to the development and worsening of psychological distress, while mental health conditions can negatively impact workplace productivity and financial decision-making stability (4–6). One promising area of work that may help mitigate financial stress is financial readiness programming (7). Financial readiness is both an individual's capacity to manage financial resources and their ability to achieve financial security (8). In this article, we use the term financial readiness to refer broadly to the knowledge, skills, confidence, and practical strategies that support individuals in managing current financial obligations, responding to unexpected financial challenges, and working toward future financial goals. Given the close link between financial stress and mental health, there remains a need to develop and test scalable and accessible approaches that integrate financial readiness programs into psychosocial interventions within the US.

One promising area within mental health research that might be well-suited for this integration is *task-sharing* (9, 10). Task-sharing is the training of trusted non-mental health specialists to deliver psychological interventions (11, 12). There now exists considerable work, within and outside of the U.S., showing that task-sharing interventions for common mental health disorders can be delivered safely, may help to overcome barriers to care, and lead to significant reductions in distress (12, 13).

This article proposes that non-specialists (e.g. staff working at community-based organizations, faith-based institutions, etc.) serving individuals in need of financial readiness programs may also be well positioned to deliver community-based mental health interventions that address both financial stress and mental health. Rather than developing distinct programs, we argue that existing evidenced-based task-sharing interventions can be adapted to address the frequently co-occurring challenges associated with financial stress and mental health. At a time when many individuals in the U.S. are experiencing both financial hardship and mental health challenges, integrating financial readiness strategies into task-sharing interventions represents a novel capacity-building approach to expanding access to care. By addressing financial stress alongside psychological distress, this integrated model has the potential to engage individuals who might not otherwise seek or receive support for either their financial or mental health needs.

To help frame why this approach may represent an important new strategy for financial stress and mental health, we begin by outlining the prevalence and factors associated with financial stress and mental health concerns in the U.S. We then summarize the evidence characterizing the bi-directional relationship between financial stress and mental health. Next, we review emerging efforts to integrate financial and mental health interventions, highlighting their potential benefits while identifying important gaps that remain in the current evidence base. The adaptation of evidence-based task-sharing interventions, such as those developed by the World Health Organization (WHO), is then presented as a promising strategy for incorporating financial-readiness content into scalable community-based mental health interventions.

## 2 Financial stress and mental health

Community-based task-sharing interventions may be well-suited to incorporate strategies that address the complex interplay between stressors linked with financial hardship and mental wellbeing. The need for such an approach is underscored by the widespread prevalence of financial insecurity in the U.S., putting many Americans in a state of financial hardship. The Consumer Financial Protection Bureau indicates that many U.S. adults experience financial insecurity, with more families reporting difficulty managing unexpected expenses nor do they feel prepared to address anticipated future costs each year (8). In fact, a recent survey found close to 40% of Americans would not have $400 in cash to pay for an emergency expense (14).

In particular, financial stress has emerged as a major consequence of financial insecurity, with nearly 50% of adults throughout the world experiencing significant stress or even chronic stress related to personal finances and inflation (15). Understanding how financial stress contributes to health symptom onset and maintenance as well as the development of effective treatments is an urgent public health matter: studies demonstrate that chronic distress is associated with reduced immune responses, cardiovascular disease, increased mortality, and economic loss (16–23). As such, considerable research has sought to elucidate how financial stress affects mental health, including associations with chronic psychological stress, material deprivation, reduced perceived control and personal agency, social exclusion, exposure to adverse living conditions, and reduced access to health-promoting resources [(5, 24, 25, 69)]. Additionally, there remain considerable barriers to mental healthcare, such as cost (26), stigma (27–29), and lack of trust in providers (30), especially for low-income and minoritized communities (31, 32).

The consequences of financial insecurity extend beyond economic hardship; financial stress has been strongly linked with a wide range of common mental health disorders. For example, studies have found that both financial burden and financial stress are correlated with depression (33–35). Although some global findings have been inconclusive on the extent to which financial stress is associated with depression [e.g., (36)], more recent reviews show positive associations between financial stress and depression across diverse contexts (37, 38). Moreover, financial hardship, perceived financial stress, debt, and worsened economic status have been linked to depression and anxiety symptoms and even reported disability (38–40). Other studies highlight how in some cases financial stress even leads to increased substance use (41–43) and in others it appears to be exacerbated by pre-existing mental health concerns [e.g., (44)].

Importantly, this relationship is also bi-directional. Individuals with mental health disorders may face several financial challenges, such as managing finances, impulsive spending, difficulty budgeting and being at risk for scams and predatory lending [e.g., (45)]. A recent genomic study in the U.K. offers some causal evidence that a mental health diagnosis such as ADHD, contributes to poverty (46). Although still emerging, a growing body of translational studies is identifying how the presence of mental health concerns may be associated with alterations in processes that guide financial decisions. For instance, Robinson and colleagues (47) found that increasing stress led to financial risks on an experimental gambling task among participants with high-trait anxiety. Relatedly, studies examining financially-relevant decision-making processes show alterations among individuals with depression, anxiety, and psychosis (48). Overall, there are likely negative cycles whereby poverty and financial stress increase psychological distress while mental health symptoms negatively impact decision-making, productivity, and economic functioning (49).

## 3 Financial strategies and stress programs

Despite the bi-directionality found between financial stress and mental health, programs and interventions aimed at ameliorating these challenges often treat them separately. For example, there are programs developed to enhance skills in financial management such as budgeting and expense tracking, debt management, goal setting, and consumer protection [e.g., (50)]. Although financial education interventions have demonstrated positive effects on financial knowledge and downstream financial behaviors, the average effects are generally small, with larger effects observed for financial knowledge than for financial behaviors (51). Moreover, there is some evidence that interventions integrating stress-reducing and mental and behavioral health components alongside the financial readiness curriculum appear to be more effective (52). Furthermore, although some financial capability programs collect data that might shed light on financial stress, the inclusion of such measures is inconsistent, highlighting a greater need for more refined approaches to address the financial stress-mental health relationship [e.g., (7)].

Some newer interventions seek to incorporate strategies aimed at reducing financial and mental distress. For example, Cook et al. (53), showed that *Building Financial Wellness* (BFW), a financial management skills program, led to significant improvements in reducing self-blame and increased proactive behavior toward finances in low-income individuals diagnosed with a psychiatric illness. Such findings suggest that integrating financial education and coaching into behavioral health services may lead to both economic and psychosocial improvements. Blanco et al. (54) conducted a randomized controlled trial (RCT) with the “*Dollars and Zen*” intervention, a community-partnered digital program that integrated financial capability training with stress-management strategies among low-income Latinx adults. Although somewhat preliminary, these findings suggest that an integrated approach may be effective in simultaneously addressing financial stress and related mental health outcomes. In addition, the *Finances First* RCT (2023) evaluated a financial education and coaching intervention designed to improve economic stability among low-income single mothers (55). The program combined financial counseling, education, and supportive coaching to address budgeting, debt management, and financial decision-making. Findings indicated reductions in financial strain alongside improvements in health-related behaviors, including decreased avoidance of medical care due to cost concerns. This suggests interventions can be developed to enhance, which in turn, may positively benefit downstream stress-related health outcomes. Another study, *TOGETHER*, examined a program integrating financial education with relationship functioning and coping skills training for couples (52). The integration of financial management (e.g., budgeting, debt communication, and planning) and relational processes, such as communication and dyadic coping, demonstrated improvements in financial capability as well as relationship functioning and psychosocial wellbeing.

## 4 Task-sharing and mental health

Although these recent studies integrating financial readiness programs with psychosocial strategies have shown promising results, these approaches could be strengthened by drawing on scalable strategies developed within the field of global mental health. Considerable evidence shows that task-sharing, the delivery of low-intensity scalable mental health and psychosocial interventions by non-specialist providers to prevent and respond to common forms of distress, plays an important role in building mental health capacity and reducing gaps in access to care (10, 11). Interestingly, however, despite the deployment of task-sharing strategies in primarily low and middle-income contexts, there are only a limited number of examples in which financially focused strategies are incorporated into broader task-sharing mental health interventions (56). The integration of financial capability elements into existing task-sharing programs represents a critical next step for enhancing scalability and sustainability of programs seeking to address both financial stress and mental health concerns. Importantly, this approach is also consistent with the WHO's stepped-care model, in which individuals receive the least resource-intense intervention appropriate for their level of need, with referral to higher-intensity care when necessary (57). An important advantage of this approach is its ability to leverage the existing non-specialist provider workforce, allowing services to reach more people without requiring a large expansion of the mental health workforce. In task-sharing interventions, non-specialists refer to individuals who are not professionally trained or credentialed as mental health specialists like psychiatrists, psychologists, or clinical social workers. Rather, they are often community-members trained and supervised to deliver specific, structured psychological interventions within clearly defined roles and referral pathways (58).

In many studies, non-specialists were staff or volunteers working for a wide range of community-based organizations or faith-based institutions that provide social services ranging from gender-based protection, food and housing security to legal and health services (59). Although task-sharing may place additional demands on non-specialist providers and may contribute to stress or burnout in some contexts, there is evidence that delivering psychosocial interventions can provide meaningful benefits to providers themselves. Non-specialists have reported applying acquired mental health skills in their personal lives, experiencing enhanced social status and community relationships, and developing a greater sense of capability and contribution through their work (10, 59).

Although many mental health studies with non-specialist providers have been developed and tested outside of the U.S [e.g., (59)], there is growing evidence that these interventions can be successfully adapted for U.S. communities (60, 61). This adaptability may be especially important given the extensive network of community-based organizations that provide social services to individuals at high risk for financial stress and mental health disorders across the United States. An important strength of task-sharing interventions is that they enable staff working in these organizations to contribute to the detection, delivery, and referral of services. As studies continue to support the training of community-based organization staff in mental health task-sharing interventions (62), these staff may also be well positioned to deliver financial stress-focused adaptations, allowing organizations to address both financial and psychological needs through a single workforce.

## 5 Integrating financial readiness and task-sharing interventions

In addition, the integration of financial readiness programs into task-sharing approaches aligns with the adaptability and flexibility of some of the most widely adopted interventions. For example, the WHO's Problem Management Plus (PM+) is designed as a brief, transdiagnostic intervention that can be delivered by non-specialist providers in diverse contexts in response to a wide range of psychosocial stressors. Similarly, Self-Help Plus (SH+) uses guided self-help materials and audio-based delivery formats that can be implemented at scale with minimal reliance on specialist personnel. Both interventions are explicitly structured to be culturally adaptable and scalable, making them particularly well suited for integration with additional content areas such as financial capability and stress management. Given that financial stress is a common and persistent driver of psychological distress, embedding financial readiness modules within these task-sharing frameworks may enhance their relevance and broaden their impact.

Task-sharing interventions such as PM+ and SH+ also have elements in their design that fit well with financial readiness programs and are thus potentially compatible given their shared emphasis on practical, skills-based strategies for managing stress and improving functioning. Both PM+ and SH+ focus on helping individuals identify key sources of distress and develop structured, actionable plans to address them through techniques such as problem-solving, behavioral activation, and stress management (58, 63). Similarly, financial readiness programs aim to strengthen individuals' ability to identify sources of financial strain and translate them into concrete action plans involving budgeting, debt management, saving behavior, and goal setting [e.g., (64)]. This overlap suggests a considerable amount of coherence as well as a similar reliance on common mechanisms, particularly the enhancement of self-efficacy and structured problem-solving in response to everyday stressors.

Important limitations to this approach need to be acknowledged. Although financial guidance and coaching may help individuals navigate financial stress and improve financial management, it is unlikely to substantially address the mental health consequences of structural poverty or severe material deprivation when individuals lack sufficient resources to meet basic needs (25, 65). Accordingly, financial guidance should be viewed as one component of a broader approach to addressing the mental health–poverty relationship, alongside interventions and policies that address income adequacy, housing, food security, employment, and other structural determinants of mental health (66, 67).

Taken together, the evidence suggests that financial stress and psychological distress cannot be effectively addressed in isolation. Although emerging interventions demonstrate promising effects on financial wellbeing, stress, and related psychosocial outcomes, they remain relatively fragmented in established global mental health delivery systems. On the other hand, task-sharing approaches such as PM+ and SH+ offer scalable, evidence-based frameworks for delivering low-intensity psychological support through non-specialist providers. The conceptual alignment between these approaches suggests a timely opportunity for integration, particularly given their shared emphasis on structured problem-solving and self-efficacy. Financial scarcity may also increase the cognitive demands of planning and decision-making, underscoring the value of manageable steps and concrete action planning when integrating financial-readiness content into task-sharing interventions (68). Embedding financial readiness components within task-sharing platforms may therefore represent a critical next step in advancing integrated, community-based interventions. We offer a conceptual case for contributing to closing the gap between two literatures: financial wellbeing and global mental health, by considering how established task-sharing platforms might be adapted to address co-occurring financial and psychological stress.

## Data Availability

The original contributions presented in the study are included in the article/supplementary material, further inquiries can be directed to the corresponding author.
